# Molecular detection of hemotropic mycoplasmas (hemoplasmas) in domestic cats (*Felis catus*) in Romania

**DOI:** 10.1186/s12917-020-02626-7

**Published:** 2020-10-22

**Authors:** Mirela Imre, Cristina Văduva, Gheorghe Dărăbuș, Sorin Morariu, Viorel Herman, Judit Plutzer, Tijana Suici, Philippa J P Lait, Kálmán Imre

**Affiliations:** 1 Department of Parasitology and Parasitic Diseases, Faculty of Veterinary Medicine, Banat’s University of Agricultural Sciences and Veterinary Medicine “King Michael I of Romania”, Timişoara, 300645 Romania; 2 Department of Internal Medicine, Faculty of Veterinary Medicine, Banat’s University of Agricultural Sciences and Veterinary Medicine Timişoara, Calea Aradului no. 119, 300645 Timişoara, Romania; 3 Department of Infectious Diseases and Preventive Medicine, Faculty of Veterinary Medicine, Banat’s University of Agricultural Sciences and Veterinary Medicine “King Michael I of Romania”, Timişoara, Romania; 4 Department of Environmental Health, National Public Health Center, 1097 Budapest, Hungary; 5 Langford Vets, University of Bristol, Langford, UK; 6 Department of Animal Production and Veterinary Public Health, Faculty of Veterinary Medicine, Banat’s University of Agricultural Sciences and Veterinary Medicine “King Michael I of Romania”, Timişoara, 300645 Romania

**Keywords:** Cats, hemotropic mycoplasmas, polymerase chain reaction, Romania

## Abstract

**Background:**

The hemotropic mycoplasmas (hemoplasmas) of the genus *Mycoplasma* are recognized as important bacteria that parasitize red blood cells, causing hemolytic anemia in many mammalian species, including cats. No information is available concerning the presence of feline hemoplasma infections in cats in Romania. Thus, the objective of the present study was to provide data on the occurrence and molecular characterization of hemotropic mycoplasmas in client-owned cats in Romania.

**Methods:**

Blood samples from 51 unhealthy cats, originating from Timişoara Municipality, Romania, were screened for the presence of hemoplasmas using conventional polymerase chain reaction (PCR) targeting the 16S rRNA gene and sequencing assays. PCR-positive samples were subsequently analyzed by phylogenetic and population genetic analysis.

**Results:**

Molecular analysis revealed 11 (21.6%) positive samples, consisting of 8 (72.7%) *Candidatus* Mycoplasma haemominutum and 3 (27.3%) *Mycoplasma haemofelis* confirmed positives. *Candidatus* Mycoplasma turicensis was not detected, and no co-infections were registered. No significant associations (*p* > 0.05) were found between the hemoplasma infection status and age, gender, breed, presence of ectoparasites, feline leukemia virus/feline immunodeficiency virus positivity of cats, or the sampling season. However, outdoor access was positively associated (*p* = 0.049) with infection and could be considered a risk factor (OR = 4.1) in acquiring feline hemotropic mycoplasmas. Phylogenetic analysis revealed that our sequences clustered with those selected from the GenBank database in two distinct clades. The registered population genetic indices were strongly supportive of the great variance in sequences between the recorded *Mycoplasma* species.

**Conclusions:**

The findings support the occurrence of feline hemoplasma infections in previously uninvestigated territories of Europe, providing useful information for small animal practitioners. To our knowledge, the present survey is the first reported molecular evidence of feline hemoplasma infections in Romania.

## Background

The hemotropic mycoplasmas (hemoplasmas) of the genus *Mycoplasma* within the Mollicutes are recognized as a class of small, wall-less, and uncultivable bacteria that parasitize red blood cells, causing hemolytic anemia in many mammalian species, including cats [1, 2].

Results of several molecular investigations conducted at a worldwide level have shown the involvement of three species in the etiology of hemoplasma infections in cats: *Mycoplasma haemofelis* (Mhf), “*Candidatus* Mycoplasma haemominutum” (CMhm), and “*Candidatus* Mycoplasma turicensis” (CMt) (reviewed by [2]). Of these, Mhf has been reported to be the most pathogenic species, being most often associated with clinical disease [3], whereas CMhm and CMt usually result in infections in individuals with concurrent retroviral, neoplastic and immune-mediated disease [1]. In addition, the zoonotic potential of some hemoplasma species has been described. Particularly, “*Candidatus* M. haemohominis”, “*Candidatus* M. haematoparvum”, *M. ovis*-like, and M. *haemofelis* have been occasionally related to human infections [4–7].

Hemoplasma infections in cats can vary in severity of symptoms, ranging from complete absence of clinical abnormalities to mild or severe, sometimes fatal acute hemolytic anemia [1]. Currently, the natural route of transmission of hemoplasmas between cats is unknown, but several modes have been suggested, including involvement of arthropod vectors (e.g., fleas, ticks) [8–11], direct transmission via blood transfusion [11], aggressive interactions between cats [12] and transplacental transmission from mother to kittens [13].

Over the last decade, the problem of continuous expansion and emergence of pathogens in novel geographical areas has attracted significant attention from researchers. In this regard, providing new data on the occurrence of unreported pathogens in an endemic area represents a priority for veterinary practitioners and the scientific community. Hemoplasma infections in cats have been molecularly confirmed in several southwestern [14–18], southern [19–21], south-eastern [22, 23], central [11, 24, 25] and northern [26] countries of Europe. However, to date, no data on the presence of feline hemoplasma infections has been reported in Romania. The present study was undertaken to address this gap by investigating the occurrence of hemoplasmas in cats from western Romania and performing the molecular characterization of the species present.

## Results

Overall, a total of 11 out of 51 domestic cats (21.6%, 95% CI 11.8–35.7) tested positive for hemoplasma by PCR amplification of the 16S rRNA gene. Two species were identified: CMhm (8/11; 72.7%) was the dominant species, and Mhf (3/11; 27.3%) was also present in a lower percentage of cats. No co-infections were registered and CMt was not detected in any of the cats. The GAPDH gene was successfully identified in blood samples of all hemoplasma-negative cats. Sequencing of PCR products was successfully performed in all positive samples. Sequence identity analysis showed that the eight CMhm and three Mhf strains derived from the present study were 98.9–100% and 97.1–100% homologous to each other, respectively. The sequences showed > 99% similarity to other GenBank-deposited CMhm (Accession no. KR905451) and Mhf (Accession no. KR905465) sequences from Italy isolated from domestic cats. Detailed distribution of the feline hemotropic mycoplasmas in accordance with the registered epidemiological data are shown in Table 1. No correlation was found (*p* > 0.05) between hemoplasma infections and the age, gender, breed, presence of ectoparasites, FeLV/FIV positivity status of cats, or the sampling season. However, cats with outdoor access (36.8%, 95% CI 17.2–61.4) were found to be more susceptible to hemoplasma infections than those without outdoor access (12.5%, 95% CI 4.1–29.9, *p* = 0.049). These results suggest that outdoor access could be considered a risk factor (OR = 4.1, 95% CI 1.0-16.6) in the acquisition of hemotropic mycoplasmas in cats.

**Table 1:** Distribution of hemoplasma infection in cats according to epidemiological data

Epidemiological data	Total no. of hemoplasma infected/tested cats (%) (95% CI)	Species identified (number)		Odds ratios (OR) (95% CI)	*p* value
		CMhm	Mhf		
Age					
Young (≤ 1-year old)	2/13 (15.4) (2.7–46.3)	0	2	1.7 (0.31–9.17)	0.533
Adult (> 1-year old)	9/38 (23.7) (12.0-40.6)	8	1		
Gender					
Female	4/28 (14.3) (4.7–33.6)	3	1	2.62 (0.7–10.5)	0.171
Male	7/23 (30.4) (14.1–53.0)	5	2		
Breed					
European	7/35 (20.0) (9.1–37.5)	4	3	1.33 (0.3–5.4)	0.687
Non-European	4/16 (25.0) (8.3–52.6)	4	0		
Sampling season					
Warm	7/31 (22.6) (10.3–41.5)	6	1	1.16 (0.3–4.6)	0.826
Cold	4/20 (20) (6.6–44.3)	2	2		
Outdoor access					
Yes	7/19 (36.8) (17.2–61.4)	5	2	4.1 (1.0-16.6)	0.049
No	4/32 (12.5) (4.1–29.9)	3	1		
Presence of ectoparasites					
Yes	3/11 (27.3) (7.3–60.7)	3	0	0.66 (0.1–3.1)	0.605
No	8/40 (20.0) (9.6–36.1)	5	3		
FeLV status					
Positive	1/5 (20.0) (1.1–70.1)	0	1	1.11 (0.1–11.1)	0.928
Negative	10/46 (21.7) (11.5–36.8)	8	2		
FIV status					
Positive	2/6 (33.3) (6.0-75.9)	2	0	2.00 (0.3–12.7)	0.462
Negative	9/45 (20.0) (10.1–35.1)	6	3		
**Total**	**11/51 (21.6) (11.8–35.7)**				

**Fig. 1:**
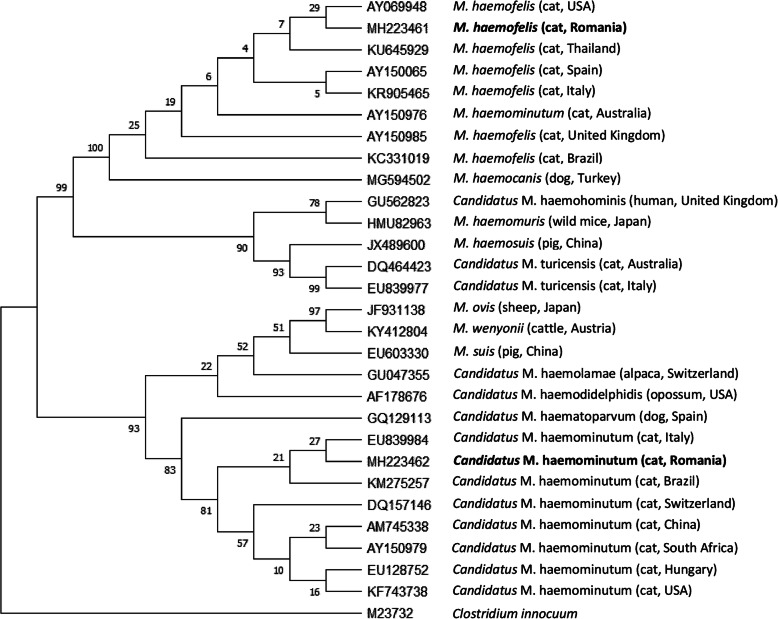
Phylogenetic tree showing the relationship of the cat origin *Mycoplasma* sequences derived from the present study (marked with bold) and other *Mycoplasma* spp. isolated from different hosts in different countries (in brackets), based on analysis of a partial sequence of the 16S rRNA gene. GenBank accession numbers are shown, and *Clostridium innocuum* (GenBank: M23732) was used as the outgroup. Numbers at branches indicate bootstrap support levels (1,000 replicates). The evolutionary history was inferred by using the Maximum Likelihood method and Tamura-Nei model [27]. The bootstrap consensus tree inferred from 1000 replicates is taken to represent the evolutionary history of the taxa analyzed [28]. Branches corresponding to partitions reproduced in less than 50% bootstrap replicates are collapsed. The percentage of replicate trees in which the associated taxa clustered together in the bootstrap test (1000 replicates) are shown next to the branches [28]. Initial tree(s) for the heuristic search were obtained automatically by applying Neighbor-Join and BioNJ algorithms to a matrix of pairwise distances estimated using the Tamura-Nei model, and then selecting the topology with superior log likelihood value. This analysis involved 29 nucleotide sequences. Codon positions included were 1st + 2nd + 3rd + Noncoding. There was a total of 538 positions in the final dataset. Evolutionary analyses were conducted in MEGA X [29]

Phylogenetic analysis based on 16S rRNA gene sequences showed the *Mycoplasma* species derived from the current survey and included in the tree construction clustered with those selected from the GenBank database. Thus, our CMhm (accession no. MH223462) and Mhf (accession no. MH223461) isolates branched separately, into one of the two resulting main clades, together with other sequences isolated from cats in Italy (accession no. EU839984) and the United States (accession no. AY069948), respectively (Fig. 1).

Results of the population genetic indices analyses performed separately for each species, as well as for all the *Mycoplasma* population sequences of 16S rRNA gene grouped together are presented in Table 2. The Tajima neutrality test yielded a value of + 2,1692, and the findings were significant (*P* < 0.05).

**Table 2:** Genetic indices of the registered cat origin 11 *Mycoplasma* sequences of 16S rRNA gene

*Mycoplasma* species	n	S	K	H	Hd ± S.D.	π ± S.D.	D
CMhm	8	3	0.70	2	0.233 ± 0.126	0.0014 ± 0.0007	-0,6544
Mhf	3	0	0.0000	1	0.0000	0.0000	0,0000
Total	11	133	56.57	3	0.5520 ± 0.089	0.1176 ± 0.0240	+ 2,1692*

*n* = number of sequences examined, *S* = number of variable sites, *K* = average number of nucleotide differences, *H* = number of haplotypes, *Hd* = haplotype diversity, π = nucleotide diversity, *S.D.* standard deviation, *D * Tajima’s D test statistics; statistical significance= * *P* < 0.05;

## Discussion

To the authors’ knowledge, this is the first molecular study documenting the occurrence of hemotropic mycoplasma infections in cats from Romania, expanding the current knowledge of feline hemoplasmas in mainland Europe.

Compared to several other European countries, the hemoplasma prevalence (21.6%) was higher than that reported in Spain (12.0%, [15]; 10.6%, [18]), Italy (18.9% [19]; 13.2%, [21]), Germany (15.6%, [24]), Denmark (16.4%, [26]) and Serbia (17.2%, [23]), similar to that described in Greece (20.6%, [22]), but lower than that reported in Portugal (27.1%, [17]; 43.4%, [16]) or northern Italy (33.1%; [20]). It is important to mention that comparing the results of different studies should be undertaken with caution, because differences in study design (e.g. sample size, sampling strategy), epidemiological parameters of the sampled population (e.g. health status of cats, cat’s living environment), and the molecular diagnostic techniques used (e.g., conventional PCR, real-time PCR, or a combination of both) in processing the blood samples in different studies could be considered sources of variation for the recorded hemoplasma prevalence. In this regard, it is important to highlight that in our study there was a bias towards the elevation of the infection prevalence, because all the investigated animals were clinically ill at presentation and sampling with suggestive signs for hemoplasma infections.

In accordance with our results, the dominance of CMhm in the screened feline population has been previously confirmed in other molecular surveys [11, 15–26], but others have reported Mhf as the predominant species [14]. It has been hypothesized previously [30] that CMHm has a more efficient replication and infection capacity but is associated with a lower pathogenic potential compared to the other two species of hemoplasma and often results in asymptomatic carriage status in cats. This can support its dominant occurrence, especially in investigations that report infections in clinically healthy cats. However, in our study considering that all the enrolled animals were clinically ill, without hematological examination, the evaluation of the pathogenicity of the recorded mycoplasma species remains an open question for future investigation.

The carriage of multiple hemoplasma species, as well as the presence of CMt was not detected. Results of other studies have shown that co-infections with different combination of hemoplasmas frequently occur [18, 21, 23], and CMt seems to be the least frequently encountered feline hemotropic mycoplasma species, with its prevalence ranging from 0.5 to 6.2% [11, 15, 16, 18, 23]. In other investigations [14, 22, 26], in which a limited number of samples were processed as in the case of our study, the lack of the CMt detection was reported. Therefore, further studies, processing a significantly larger number of samples, are still necessary to obtain a more accurate overview and to conclude whether CMt is implicated in Romanian cats’ mycoplasma infections.

Outdoor access was the only epidemiological measure that was associated with hemoplasma infection in this study. Other studies have described several factors significantly associated with the presence of hemotropic mycoplasmas in cats, including adult [18] or older [11, 22, 24–26, 30] age, male gender [11, 18, 21, 23–25, 30], non-pedigree breed [23], collection of blood during warm months [18, 19], and FeLV/FIV positivity status [15–19, 21, 23–25, 30]. Similar to our finding, the increased likelihood of cats being infected with hemotropic mycoplasmas with outdoor access has been frequently reported in other studies [11, 15, 18, 23]. This observation can be sustained by the fact that this lifestyle increases, diversifies and perpetuates the relationships between cats, resulting in the possible transmission of mycoplasma from positive to negative animals, via direct (e.g., fighting or biting) or vector-borne (fleas or ticks) transmission, as have been previously suggested [8–12, 15, 21, 30]. However, until now, the scientific demonstration of this hypothesis has remained, until now, unfulfilled.

The structure of the constructed tree, based on 16S rRNA gene phylogeny, demonstrated that our isolates were grouped in distinct clades, together with other GenBank-deposited *Mycoplasma* sequences isolated from cats in different countries from various parts of the world. This phenomenon has been previously observed by Tasker et al. [31]. However, it is important to note that the evolutionary distance analysis indicate the same bootstrap support level between Mhf and *M. haemocanis* and very similar between CMt and *Candidatus* M. haematoparvum. The observed unreliable division between these different host-specific mollicutes suggests the use of a more reliable and representative marker in supporting the existence of genetic differences between hemoplasmas infecting cats and dogs [31].

The relatively high values of the majority (S, K, Hd) from the registered total population genetic indices (Table 2), together with the significantly positive value of the Tajima neutrality test, are strongly supportive of the great variance in sequences between the recorded two-cat origin *Mycoplasma* species. In the case of CMhm, the obtained low nucleotide diversity (π) value is suggestive of the occurrence of two closely related haplotypes which differ in a small number of nucleotides. Also, the observation of a total high haplotype and relatively low nucleotide diversity within the *Mycoplasma* population may suggestive for a possible population genetic expansion pattern in the future, as has been previously reported [32].

## Conclusions

The present study provides data on the occurrence of hemoplasma infections as well as the molecular evidence of CMHm and Mhf supported by phylogenetic and population genetic analysis in domestic cats for the first time in Romania. As such, it provides the first indication of the prevalence of hemoplasmas in this previously uninvestigated territory and useful information for small animal practitioners. It implicates the outdoor access lifestyle as a risk factor in the acquisition of disease. The occurrence of feline hemoplasmas in this geographical area, previously thought to be hemoplasma free, opens the opportunity for a larger scale study to be carried out to address some of the limitations of the current survey. In this regard, further studies focusing on the relationship between the hemoplasma species and the resultant hematologic profile, with special emphasis on anemia are recommended.

## Methods

### Sample collection and anamnesis

From April 2017 to February 2019, a total of 51 client-owned cats originating from Timişoara Municipality were presented at the Veterinary Clinics of the Faculty of Veterinary Medicine in Timisoara, Romania, for medical consultation showing one or more suggestive clinical signs (e.g., pallor of the mucous membranes, intermittent pyrexia, weight loss, lethargy, dehydration, or weakness) for feline hemoplasma infections. On the day of presentation, in addition to routine physical examination, all cats were screened for feline leukemia virus (FeLV) and feline immunodeficiency virus (FIV) status using a commercial kit (IDEXX Laboratories, Inc., Westbrook, Maine, USA). In addition, blood was collected from each cat into sterile vacuum tubes with ethylenediaminetetraacetic acid (EDTA) from the antebrachial cephalic vein, to test the presence of hemoplasma DNA. During anamnesis, owners provided information about the cat, including age, gender, breed, outdoor access and any history of previous ectoparasites (i.e., ticks and/or fleas) infestation were collected during anamnesis.

### Molecular diagnosis

As polymerase chain reaction (PCR) assays provide a far more specific and sensitive approach to hemoplasma identification than cytology of blood -smears [2], all biological samples were directly subjected to molecular processing. Genomic DNA was isolated using a PureLink™ genomic DNA mini kit (Invitrogen™, Carlsbad, CA, USA) according to the manufacturer’s instructions. Detection of feline hemotropic mycoplasmas was carried out using conventional PCR based on the amplification of a partial sequence of the 16S rRNA gene. The specific forward (5′-ACGAAAGTCTGATGGAGCAATA-3′) and reverse (5′-ACGCCCAATAAATCCGRATAAT-3′) primers and cycling parameters were used as previously described by [33]. These primers produce a 193 bp amplified fragment in CMhm and 170 bp in Mhf or CMt. Next, differentiation between Mhf and CMt in PCR-positive samples was carried out using a second PCR protocol with the CMt-specific forward (5′-AGAGGCGAAGGCGAAAACT-3′) and reverse (5′-CTACAACGCCGAAACACAAA-3′) primers and cycling conditions according to [30, 34]. Subsequently, to obtain a longer *Mycoplasma* sequence, and for a better molecular assessment of infections in cats, a third conventional PCR was achieved for the previously obtained PCR-positive results, using the universal HBT-forward (5′-ATACGGCCCATATTCCTACG-3′) and HBT-reverse (5′-TGCTCCACCACTTGTTCA-3′) primer set and cycling conditions designed by [14]. The positive PCR reactions produce a 595 bp and 618 bp long amplicons for Mhf and CMhm, respectively. CMhm (reference sequence AF271154) and Mhf (reference sequence AF178677) DNA isolated from blood of naturally infected cats (Diagnostic Laboratories, Langford Vets, University of Bristol, UK), previously confirmed by sequencing, was used as the positive control. The negative control consisted of sterile deionized water. In hemoplasma negative DNA samples, an internal control assay was run targeting the housekeeping feline glyceraldehyde 3-phosphate dehydrogenase (GA3PDH) gene, to ensure the presence of the amplifiable DNA, as well as the absence of PCR inhibitors. The PCRs were performed as described previously [35] using the GAPDH – forward (5′-CCTTCATTGACCTCAACTACAT-3′) and GAPDH – reverse (5′-CCAAAGTTGTCATGGATGACC-3′) specific primer set.

All PCR products were visualized on Midori Green (Nippon Genetics®; Europe, Gmbh) stained 1.8% agarose gel.

The PCR amplified 16S rRNA gene amplicons showing positive results were purified (Isolate II PCR and Gel Kit, Bioline™) and bidirectionally sequenced (performed by Macrogen™ Europe, Amsterdam, the Netherlands). The resulting sequences were analyzed for their homology using the Clustal Omega (available online: https://www.ebi.ac.uk/Tools/msa/clustalo/) multiple sequence alignment program and subjected to BLAST (Basic Local Alignment Search Tool) analysis to compare them to those available in the GenBank™ dataset. All the obtained sequences were deposited into GenBank™ as follows: Mhf: MH223461 and MT926037 - MT926038; and CMhm: MH223462 and MT926039 - MT926045.

### Phylogenetic and population genetic analysis

Phylogenetic analysis was performed with MEGAX (ver. 10.1) software [29]. The evolution of two representative DNA sequences from the present study and those available from the GenBank database were measured by the Tamura-Nei model [27]. The tree was constructed using the maximum likelihood method with 1000 bootstrap replicates. The characterization of the bacterial strain population diversity indices, including the nucleotide diversity (π), haplotype diversity (Hd), number of variable sites (S), and nucleotide differences (K), was carried out using the DnaSP (ver. 6.12) software [36]. In addition, the Tajima neutrality test was also computed [37].

### Statistical analysis

Statistical analyses were performed using SPSS 21.0 software. The possible association between the hemoplasma infection status of cats and the recorded epidemiological parameters was assessed with the nonparametric Pearson’s chi – squared (χ2) test. Differences were established as statistically significant when *p* value ≤ 0.05. Also, the risk factors were evaluated through calculation of odds ratios (ORs) with 95% confidence intervals (CIs) by including each variable in the binary Logit model of the multivariate regression analysis.

## Data Availability

The sequences (accession numbers MH223461, MT926037 - MT926038, MH223462, and MT926039 - MT926045) used to support the findings of this study have been deposited in the GenBank repository. The datasets generated and analyzed during the current study are included within the article.

## Abbreviations

Mhf: *Mycoplasma haemofelis*
CMhm: *Candidatus* Mycoplasma haemominutum
CMt: *Candidatus* Mycoplasma turicensis
FeLV: feline leukemia virus
FIV: feline immunodeficiency virus
DNA: deoxyribonucleic acid
EDTA: ethylenediaminetetraacetic acid
PCR: polymerase chain reaction
RNA: ribonucleic acid
BLAST: Basic Local Alignment Search Tool
OR: odds ratio
CI: confidence interval
